# Comparative study between photodynamic therapy and the use of probiotics in the reduction of halitosis in mouth breathing children: Study protocol for a randomized controlled clinical trial

**DOI:** 10.1097/MD.0000000000033512

**Published:** 2023-04-14

**Authors:** Laura Hermida Bruno, Ana Paula Taboada Sobral, Marcela Leticia Leal Gonçalves, Ana Laura Fossati, Elaine Marcilio Santos, Juliana Maria Altavista Sagretti Gallo, Elza Padilha Ferri, Pamella de Barros Motta, Renato Araujo Prates, Alessandro Melo Deana, Anna Carolina Ratto Tempestini Horliana, Lara Jansiski Motta, Sandra Kalil Bussadori

**Affiliations:** a Postgraduation Program in Biophotonics Applied to Health Sciences, Universidade Nove de Julho, São Paulo, Brazil; b Universidad Católica del Uruguay (UCU), Montevideo, Uruguay; c School of Dentistry, Universidade Metropolitana de Santos, Santos, Brazil; d Postgraduation Program in Health and Environment, Universidade Metropolitana de Santos, Santos, Brazil; e Postgraduation Program in Veterinary Medicine in the Coastal Environment, Universidade Metropolitana de Santos, Santos, Brazil.

**Keywords:** halitosis, mouth breather, photodynamic therapy, probiotics

## Abstract

Halitosis is a term that defines any odor or foul smell the emanates from the oral cavity, the origin of which may be local or systemic. One of the causes of local or oral halitosis is low salivary flow and dry mouth, which is also one of the complaints of individuals with the mouth-breathing habit. The aim of this study is to determine the effectiveness of antimicrobial photodynamic therapy (aPDT) and the use of probiotics for the treatment of halitosis in mouth-breathing children.

**Methods:**

Fifty-two children between 7 and 12 years of age with a diagnosis of mouth breathing and halitosis determined through an interview and clinical examination will be selected. The participants will be divided into 4 groups: Group 1—treatment with brushing, dental floss and tongue scraper; Group 2—brushing, dental floss and aPDT applied to the dorsum and middle third of the tongue; Group 3—brushing, dental floss and probiotics; Group 4—brushing, dental floss, aPDT and probiotics. The use of a breath meter and microbiological analysis of the tongue coating will be performed before, immediately after treatment and 7 days after treatment. The quantitative analysis will involve counts of colony-forming bacteria per milliliter and real-time polymerase chain reaction. The normality of the data will be determined using the Shapiro–Wilk test. Parametric data will be submitted to analysis of variance and nonparametric data will be compared using the Kruskal–Wallis test. The results of each treatment in the different periods of the study will be compared using the Wilcoxon test.

**Discussion:**

Due to the low level of evidence, studies are needed to determine whether treatment with aPDT using annatto as the photosensitizer and blue led as the light source is effective at diminishing halitosis in mouth-breathing children.

## 1. Introduction

Halitosis is a term that defines any odor that emanates from the oral cavity, the origin of which may be local or systemic.^[1]^ The odor may be attributed to a variety of products resulting from the metabolism of bacterial amino acids. Halitosis is classified as genuine, pseudo-halitosis and halitophobia. Genuine halitosis is divided into physiological halitosis (caused mainly by coated tongue) and pathological halitosis, which can be oral (oral disease) or extraoral (systemic diseases). Pseudo-halitosis is self-perceived by the individual even when not perceived by others and not diagnosed clinically. Halitophobia is a condition in which the patient complains of bad breath even with no clinical or social evidence and after specific treatments.^[2]^

The prevalence of halitosis is high, with the literature reporting rates of more than 50%.^[3]^ This condition is considered an important social factor that interferes with interpersonal relationships. Besides causing concerns related to physical health, halitosis can be a social barrier and lead to psychological problems.^[4]^ In this context of social and biological importance, the prevalence of halitosis and associated factors in the pediatric population has been investigated throughout the world and different rates are estimated.^[1,5–10]^

Recent studies involving mouth-breathing children have demonstrated that this group has a significantly greater frequency of halitosis compared to nose breathers.^[8,11–13]^ Halitosis classified as oral originates in the mouth and upper airways and results from the decomposition of organic matter—epithelial cells are retained in the posterior portion of the tongue due to low salivary flow and/or a water imbalance as well as microbial attack in the oral environment, which favor the growth of proteolytic bacteria, resulting in the production of volatile sulfur compounds, which are related to the characteristic odor.^[14,15]^ When salivary flow rate is low, the bacterial count and halitosis in the oral cavity increase. The change from nose breathing to mouth breathing causes changes in the dental arches and surrounding tissues, such as anatomic changes in the palate and the drying of the surface of the mucosa, which is one of the main complaints of mouth breathers and may be related to halitosis.^[3,16]^ Individuals who breath through the mouth due to adenotonsillar hypertrophy have higher rates of halitosis compared to treatment groups submitted to surgery and control groups (nose breathers).^[10]^

Volatile sulfur compounds (VSCs) are related to the occurrence of halitosis. Hydrogen sulfide is associated with coated tongue, methyl mercaptan is associated with periodontal pockets and dimethyl sulfide is associated with systemic alterations.^[5,17–19]^ Among the different methods for the diagnosis of halitosis, the clinical examination, known as the organoleptic test, is a subjective method consisting of smelling the odor exhaled from the mouth and nose and quantifying this odor with the use of a scale. VSCs can be measured with the use of sulfide monitors and gas chromatography.^[2,5,11,20]^ The portable Breath-Alert device (BA) has been used with increasing frequency in clinical practice for the diagnosis of halitosis due to its ease of use and low cost.^[16,21–25]^ In children, who need fast, practical exams, the BA is a halitosis detection tool with high sensitivity and specificity for use in pediatric dentistry.^[8]^

Conventional treatments used in the control of halitosis consist basically of the use of toothpastes and mouthwashes containing bactericidal substances, a tongue scraper, the treatment of dental caries and periodontal disease and the control of xerostomia.^[26]^ Some studies suggest that amine fluoride has a positive effect on diminishing halitosis.^[3]^ Alternative treatments, such as antimicrobial photodynamic therapy (aPDT)^[10,21,27]^ and probiotics, have also been employed in an attempt to control this condition.^[3,16,22,23,26]^ Antimicrobial PDT is treatment in which a photosensitizing agent (dye) produces oxygen free radicals in the presence of light, leading to bacterial cell death. As the main etiological factor of halitosis is the presence of anaerobic bacteria, aPDT (red laser and methylene blue) has demonstrated positive results on the reduction of hydrogen sulfide as well as the bacterial load on the dorsum of the tongue.^[15,21]^ The dye annatto has been evaluated as a photosensitizer in studies on halitosis. This extract from the seeds of the plant Bixa orellana, which is native to Brazil, is accepted by the World Health Organization due to the fact that it is nontoxic.^[1]^ Annatto has antioxidant and antimicrobial properties^[9]^ and recent studies have demonstrated its potential as a therapeutic agent and natural dye.^[28]^

Probiotics are microorganisms that provide health benefits when absorbed by the host and are often used in foods and fermented products as well as pharmaceutical formulations.^[25]^ Studies have shown the positive results of probiotics for the control of halitosis and suggest that these products favor the elimination of undesirable microorganisms and promote the recolonization of the microbiota.^[16,22]^

Alternatives such as aPDT with annatto and the administration of probiotics for the reduction in or elimination of halitosis in pediatric dentistry are less invasive methods that involve natural components, which reduces harm to oral tissues and avoids the occurrence of bacterial resistance. The major challenge is the development of an effective, lasting treatment protocol for halitosis in children that eliminates the anaerobic bacteria related to this condition and reestablishes the microbiota of the dorsum of the tongue, thereby improving quality of life. The use of probiotics in dentistry constitutes innovative treatment capable of modifying the oral microbiota as an alternative to the use of antibiotics and other antimicrobial agents.

The treatment of halitosis is a topic that requires greater attention, and the results of this study can assist in clinical decision making with regards to the use of probiotics and aPDT with blue light-emitting diodes (LEDs) for the treatment of this condition, as most dentists have this light source in their offices and the portable sulfide meter is inexpensive to acquire. Moreover, the use of annatto as a photosensitizer is an innovative approach. As the light source and photosensitizer are accessible, this treatment is expected to be easily and effectively reproducible clinical practice. Probiotics and aPDT are expected to be effective at diminishing halitosis in mouth-breathing children.

The main of the study is to determine whether treatment with aPDT using annatto as the photosensitizer and blue led as the light source is effective at diminishing halitosis in mouth-breathing children.

## 2. Methods

### 2.1. Study registration

This is the first version of a protocol that was registered in ClinicalTrials.gov, under the registration number: NCT05590897, first posted on 10/21/2022 and last updated on 10/21/2022. Available online at: https://clinicaltrials.gov/ct2/show/NCT05590897.

### 2.2. Type of study

**Figure 1.**
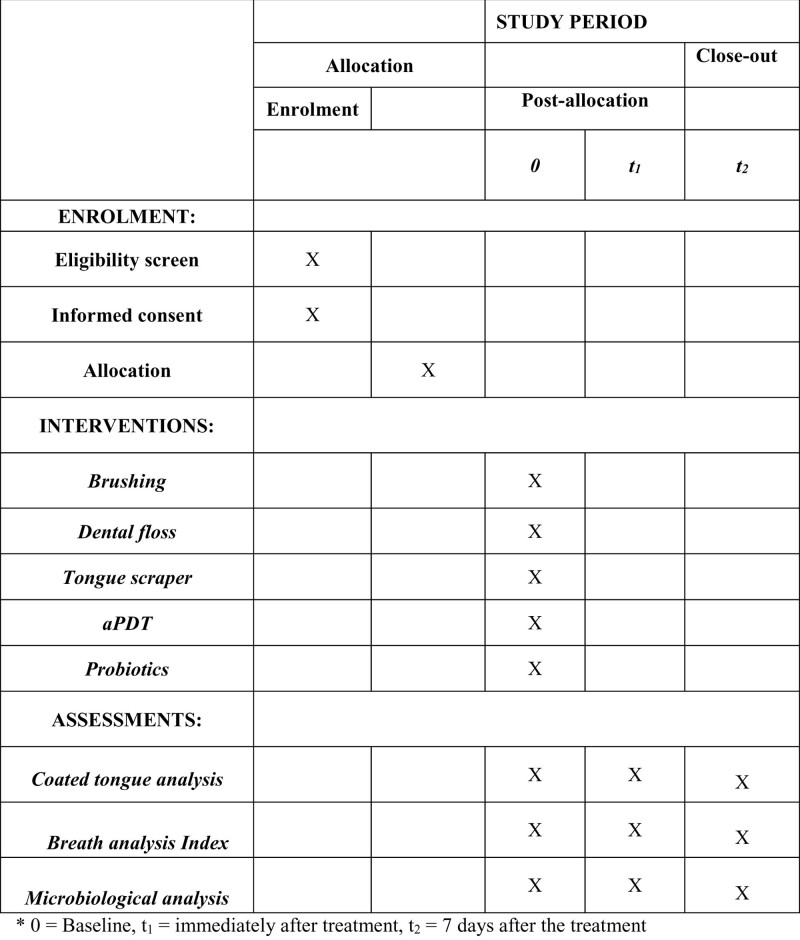
SPIRIT figure as recommended by 2013 SPIRIT statement.

A randomized, controlled clinical trial was designed based on the Consolidated Standards of Reporting Trials statement. It is described according to the criteria established in the Standard Protocol Items: Recommendations for Interventional Trials statement and Figure 1 is the Standard Protocol Items: Recommendations for Interventional Trials figure.

### 2.3. Ethics committee

The study will be conducted in accordance with the ethical precepts stipulated in the Declaration of Helsinki (World Medical Association Declaration of Helsinki, 2008). The protocol for this study was approved by the Human Research Ethics Committee of Universidade Metropolitana de Santos-UNIMES (certificate number: 64510922.6.0000.5509). All information will be contained in the statement of informed consent in accordance with Resolution 196 of the National Board of Health (Health Ministry, Federal District, Brazil, March 10, 1996). The guardians of the children will agree to participate by signing a statement of informed consent; 2 copies of which will be signed—one for the guardian and one for the researchers.

The participants will be informed that they may withdraw from the study at any time for any reason, if they so wish. The researchers will also be able to remove participants from the study if deemed necessary.

### 2.4. Participants

The study will include Male and female children 7 to 12 years of age who enter the dental clinic of Universidade Metropolitana de Santos (UNIMES), for treatment. At the first appointment, a form addressing the medical history of the patient will be completed. The volunteers will then be submitted to a clinical examination for the determination of oral health status. Next, the inclusion and exclusion criteria will be applied.

#### 2.4.1. Inclusion criteria.

Children and adolescents with a diagnosis of mouth breathing determined using a standard questionnaire and halitosis determined using a portable breath meter (score ≥ 2) will be included in the study.

Parents/guardians will provide data on family income, parent’s schooling, child’s previous experience with dental treatment, the occurrence of snoring, open mouth during sleep, daytime sleepiness, saliva on the pillow during sleep, awaking with thirst during the night, dry mouth, nocturnal enuresis, headache, aggressivity, inattention, hyperactivity, abnormal respiration, swallowing difficulty, sense of smell and taste, speaking, negative effect on school performance and the presence of allergies. Parents/guardians will also report their child’s toothbrushing frequency, their participation in brushing, the child’s eating habits, child’s toothpaste, the occurrence of poor oral habits, such as bruxism, nail biting, lip biting, tongue bite, tongue pushing, smacking the lips and whether they have detected bad breath in their child or themselves.

#### 2.4.2. Exclusion criteria.

Nose breathers, individuals with dentofacial anomalies (e.g., hare lip and cleft palate), those in orthodontic and/or orthopedic treatment, those in oncological treatment, those with systemic (gastrointestinal, renal or hepatic) conditions, pregnant girls, individuals having undergone antibiotic treatment in the previous month and individuals with fissured tongue will be excluded.

### 2.5. Patient and public involvement

The guardians of the patients were not involved in the design of this study. After the data analysis, the guardians will be given the opportunity to participate in a result-sharing meeting if they so desire. The consent form signed by guardians of the participants explains that the storage of data for each participant and family member is within the terms of confidentiality.

### 2.6. Calculation of sample size

The sample size will be calculated using data from the study by Costa da Mota et al.^[21]^ An error $err=\left|\underline{x_{1}}-\underline{x_{2}}\right|$ was first established, in which $\underline{x_{1}}$ and $\underline{x_{2}}$ are the means of the groups at baseline for periodontal treatment with PDT. The effect size was calculated based on this error as follows:


$$\begin{array}{l} \frac{err}{\sqrt{\sigma_{1}^{2}+\sigma_{2}^{2}}} \end{array}$$

in which $\sigma_{1}^{2}$ and $\sigma_{2}^{2}$ are the variances of Groups 1 and 2, respectively.

**Figure 2.**
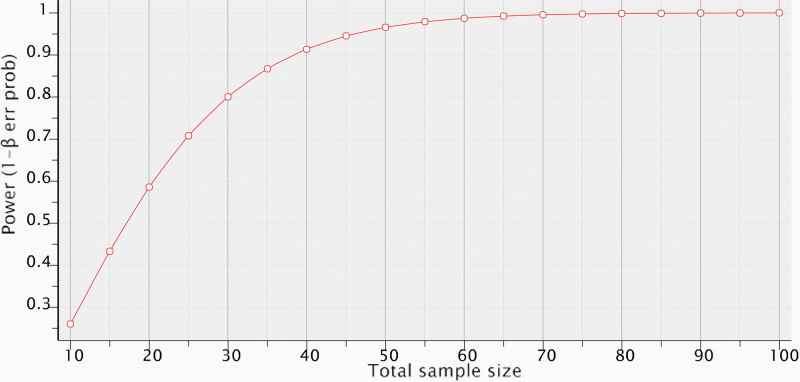
Adjustment of test power as function of total sample size.

Assuming that the groups studied have normal or approximately normal distribution, that the sample size will be sufficiently large and that a 2-tailed test will be used for a significance level of α = 0.05 and maintaining a test power of 1 − β = 0.90, n = 13 will be needed for each group. Figure 2 shows that a sample of 52 children (4 groups of 13 individuals) would enable demonstrating statistically significant differences while maintaining a test power equal to or higher than 0.90. If the hypothesis of normality of the distributions is rejected, the sample size should be corrected by approximately 5%.

### 2.7. Randomization

The type of treatment will be determined randomly for each patient prior to the intervention. Randomization will be generated electronically using the randomizer.org site for the balanced distribution of the children among the groups.

Fifty-two mouth-breathing children with a diagnosis of halitosis will be selected and divided into 4 groups.

Group 1: Treatment with brushing, dental floss and tongue scraper;

Group 2: Brushing, dental floss and aPDT applied to the dorsum and middle third of the tongue;

Group 3: Brushing, dental floss and probiotics;

Group 4: Brushing, dental floss, aPDT and probiotics.

Breath meter results will be determined before, immediately after treatment and 7 days after treatment. Microbiological analysis of the tongue coating will performed at the same times. The quantitative analysis will be performed using direct plating and real-time polymerase chain reaction (PCR).

### 2.8. Interventions

#### 2.8.1. Coated tongue analysis.

The quantity of tongue coating will be defined using the Coated Tongue Index proposed by Shimizu et al.^[29]^ The tongue is divided into 9 parts, each of which receives a score: 0—absence of coated tongue, 1—thin tongue coating with visible papillae, 2—thick tongue coating, papillae not visible.

#### 2.8.2. Breath analysis.

**Figure 3.**
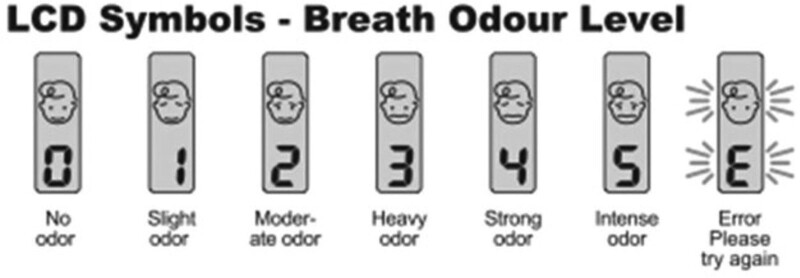
Breath odor analysis with breath meter.

Air from the oral cavity will be collected following the manufacturer’s orientations. The BA will be disinfected after each use. The device will be shaken 4 or 5 times prior to use to eliminate any residual odors. A “beep” is emitted upon opening the upper compartment of the device and a second “beep” is emitted when the volunteer blows into the front air input (passage of airflow). After a third “beep,” the odor of the breath is measured and scored on a scale of 0 to 5 points. An “E” appears when an error has occurred and the procedure is repeated (Fig. 3). A score of ≥2 points is considered indicative of halitosis.

To avoid the influence on the breath analysis results, the participants will be instructed to obey the following: in the 48 hours prior to the evaluation, avoid foods with garlic, onion, strong spices, the consumption of alcohol and the use of an oral antiseptic; on the day of the evaluation, abstain from all food in the 2 hours prior to the exam, abstain from coffee, mints, chewing gum, oral and personal hygiene products with perfume (aftershave, deodorant, perfume, creams and tonic) and brush only with water.

#### 2.8.3. Microbiological analysis.

Microbiological collections will be performed before and immediately after treatment. Samples of the tongue coating will be collected with a swab, soaked in reduced Amies medium. The samples will be taken to the laboratory for analysis in less than 2 hours after collection. The samples will be vortexed for approximately 1 minute. After homogenization, 10 serial dilutions will be prepared in 180 μL of sterile phosphate buffered saline. Aliquots of 10–1, 10–2, 10–3, 10–4, and 10–5 will be transferred to Petri dishes with sheep blood agar. As the main bacteria responsible for the production of VSCs are anaerobic, the Petri dishes will be incubated in anaerobiosis for 72 hours at 37°C. Bacterial counts will be based on colony-forming units.^[4,21]^

#### 2.8.4. Microbiological analysis—real-time PCR.

The samples of coated tongues will be collected with a sterile swab that will be placed in contact with the surface of the dorsum of the tongue with back-and-forth movements. The samples will be placed in tubes containing Tris–EDTA buffer (10 mM Tris—HCL, 0.1 mM EDTA, pH 7.5). The tubes will be duly labeled and stored at −80 °C until analysis. Maximum storage time will be 1 week. The samples will be frozen due to the impossibility of performing all analyses on a single day. After thawing, the samples will be vortexed for 1 minute. The Master Pure DNA Extraction kit (Epicentre Technologies, Chicago, IL) will be used for the extraction of bacterial DNA. First, 100 μL of sample will be eluted in Tris-EDTA buffer. The samples will be centrifuged at 10,000 rpm and 4 °C for 10 minutes. Next, the MIX will be prepared by diluting 300 µL of TCL + 2 µL of Prot K for each sample. The supernatant will be removed with a pipette, with a volume of 25 µL + the formed pellet remaining. Vortexing will be performed for 10 seconds to resuspend the pellet. A total of 302 µL of MIX will be added to each sample, followed by vortexing for 10 seconds and incubation at 65 °C for 15 minutes with vortexing every 5 minutes. The samples will be placed in ice for 5 minutes. A total of 150 µL of MPC (protein precipitation reagent) will be added to the samples, followed by vortexing for 10 seconds. The samples will be centrifuged at 10,000 rpm and 4 °C for 10 minutes. The supernatant will be removed and placed in a new tube, to which 25 µL of MPC (protein precipitation) will be added. The tube will be centrifuged at 10,000 rpm and 4 °C for 10 minutes. A total of 500 µL of isopropanol will be added and inverted 40×, followed by centrifugation at 10,000 rpm and 4 °C for 10 minutes. The samples will be placed in ice without releasing the pellet. The isopropanol will be poured out and the rest will be removed with a 50-µL pipette. Washing will be performed with 200 µL of 70% ethanol carefully so as not do dislodge the pellet. Centrifugation will be performed at 10,000 rpm and 4 °C for 3 minutes. The residual ethanol will be removed and the sample will be left inverted to dry for 30 minutes. Elution will be performed in 40 µL of Tris-EDTA buffer, followed by freezing at −20 °C. The purified DNA will be resuspended in Tris-EDTA buffer. Levels of *Porphyromonas gingivalis, Tannerella forsythia*, and *Treponema denticola* will be determined by quantitative PCR. The quantitative analysis will be performed by real-time PCR using a Step One Plus Real-Time PCR System thermocycler (Applied Biosystem, Foster City, CA) and the products will be detected by fluorescence using the Quantimix Easy SYG Kit (Biotools, Madrid, Spain), following the manufacturer’s protocol. For the reaction, 10 µL of SYBR Green, 0.5 µL of DNA mold and 200 mM of each primer (*P gingivalis* CATAGATATCACGAGGAACTCCGATT and AAACTGTTAGCAACTACCGATGTGG; *T forsythia* GGGTGAGTAACGCGTATGTAACCT and ACCCATCCGCAACCAATAAA; *T denticola* CGTTCCTGGGCCTTGTACA and TAGCGACTTCAGGTACCCTCG; and the universal primers for bacteria CCATGAAGTCGGAATCGCTAG and GCTTGACGGGCGGTGT) will be used in a total volume of 20 µL. Sterile Milli-Q water rather than DNA mold will be used as the negative control. Reactions for 16S rRNA will be performed with initial denaturation at 95 °C for 2 minutes, followed by 36 cycles of 94 °C for 30 seconds, 55 °C for 1 minute and 72 °C for 2 minutes and final extension at 72 °C for 10 minutes. Fluorescence will be detected after each cycle and represented in a graph using the Step One Plus Real-Time PCR System (Applied Biosystem). To ensure the specificity of the products detected by fluorescence and avoid the detection of primer dimers, detection will be performed 1 degree below the dissociation temperature of the amplicons. All samples will be analyzed in duplicate and each dilution of plasmids for the standard curve will be analyzed in triplicate.^[3]^ The purpose of the microbiological analysis will be to determine the effectiveness of photodynamic therapy for the treatment of halitosis, complementing the clinical assessment.

#### 2.8.5. Antimicrobial photodynamic therapy.

The Valo Cordless Ultradent® LED curing light will be used. This device has a coupled radiometer, spectrum of 440 to 480 nm and irradiance of 450 mW/cm. Only the volunteer being treated, and operator of the device will be present at the time of aPDT, both of whom will be using protective eyewear. The active LED tip will be covered with disposable transparent plastic wrap (PVC) to avoid cross-contamination and for hygiene purposes and the operator will be duly garmented.

One session of aPDT will be performed with the annatto photosensitizer at a concentration of 20% (Fórmula e Ação®) in spray form to be applied in a sufficient quantity to cover the middle third and dorsum of the tongue (5 sprays) with 2 minutes for incubation. The excess will be removed with an aspirator while maintaining the surface moist with the photosensitizer without the use of water. Six points will be irradiated with a distance of 1 cm between points, considering the light scattering halo and effectiveness of aPDT. The device will be previously calibrated with a wavelength of 395 to 480 nm, 20 seconds per point and energy of 9.6 J. The light will be irradiated such that a halo will be 2 cm in diameter per point. Table 1 displays the parameters that will be used.^[30]^

**Table 1:** LED parameters.

Wavelength (nm)	395–480
Operating mode	Continuous
Mean radiant power (mW)	480
Polarization	Random
Aperture diameter (cm)	0.9
Irradiance at aperture (mW/cm^2^)	762
Beam profile	Top hat
Irradiated area (cm^2^)	3.14
Irradiance at target (mW/cm^2^)	153
Exposure time (s)	20
Radiant exposure (J/cm^2^)	6.37
Radiant energy (J)	9.6
Number of points irradiated	6
Area irradiated (cm^2^)	18.8
Number and frequency of treatment sessions	1
Total radiant energy (J)	57.6

LED = light-emitting diode.

#### 2.8.6. Tongue scraping.

Tongue scraping will be performed by the same operator on all participants using posteroanterior movements of the scraper on the dorsum of the tongue, followed by cleaning of the scraper with gauze. This procedure will be performed 10 times on each patient to standardize the mechanical removal of the tongue coating.

#### 2.8.7. Treatment with probiotics.

Pharmaceuticals (capsules or chewable gum) will be prepared at a compounding pharmacy. The products will contain strains of Lactobacillus salivarius WB21 (6.7 × 108 colony-forming units) and xylitol (280 mg). Each patient should take the pharmaceutical 3 times per day after meals for 7 days.

#### 2.8.8. Brushing with toothpaste containing amine fluoride.

All 52 participants will be instructed to brush with a toothpaste containing amine fluoride (Elmex®) and use dental floss 3 times per day after meals for 10 days.

### 2.9. Statistical analysis

Data from the portable Breath-Alert™ device will be analyzed for normality using the Shapiro–Wilk test. Parametric data will be submitted to analysis of variance followed by Tukey’s test when necessary and the *t* test will be used for paired data to analyze the results of treatment in the 2 periods of the study. Nonparametric data will be compared using the Kruskal–Wallis test followed by the Student–Newman–Keuls test when necessary and the Wilcoxon text will be used for the comparison of each treatment in the 2 periods of the study.

## 3. Discussion

The objective of the present study is to determine whether treatment with aPDT using annatto as the photosensitizer and blue led as the light source is effective at diminishing halitosis in mouth-breathing children. Through this study, we will be able to determine whether there are differences among the proposed treatments. We will also determine whether a reduction in halitosis occurs after the use of photodynamic therapy involving the use of annatto as the photosensitizer and blue LED as the light source and after treatment with probiotics.

## Acknowledgments

The authors are grateful to Universidade Metropolitana de Santos (UNIMES) for the availability of laboratories and volunteers. To the guardians of the patients for agreeing to participate in this study.

## Author contributions

**Conceptualization:** Laura Hermida Bruno, Ana Paula Taboada Sobral, Elaine Marcilio Santos, Pamella de Barros Motta, Lara Jansiski Motta, Sandra Kalil Bussadori.

**Data curation:** Renato Araujo Prates, Alessandro Melo Deana, Anna Carolina Ratto Tempestini Horliana, Lara Jansiski Motta.

**Formal analysis:** Laura Hermida Bruno, Alessandro Melo Deana, Anna Carolina Ratto Tempestini Horliana, Lara Jansiski Motta, Sandra Kalil Bussadori.

**Investigation:** Laura Hermida Bruno, Sandra Kalil Bussadori.

**Methodology:** Laura Hermida Bruno, Ana Paula Taboada Sobral, Marcela Leticia Leal Gonçalves, Renato Araujo Prates, Sandra Kalil Bussadori.

**Project administration:** Laura Hermida Bruno, Sandra Kalil Bussadori.

**Software:** Juliana Maria Altavista Sagretti Gallo, Renato Araujo Prates, Alessandro Melo Deana, Anna Carolina Ratto Tempestini Horliana, Lara Jansiski Motta.

**Supervision:** Laura Hermida Bruno, Ana Paula Taboada Sobral, Alessandro Melo Deana, Sandra Kalil Bussadori.

**Validation:** Laura Hermida Bruno, Marcela Leticia Leal Gonçalves, Renato Araujo Prates, Alessandro Melo Deana, Anna Carolina Ratto Tempestini Horliana, Sandra Kalil Bussadori.

**Visualization:** Laura Hermida Bruno, Ana Paula Taboada Sobral, Ana Laura Fossati, Sandra Kalil Bussadori.

**Writing – original draft:** Laura Hermida Bruno, Ana Paula Taboada Sobral, Marcela Leticia Leal Gonçalves, Ana Laura Fossati, Elaine Marcilio Santos, Juliana Maria Altavista Sagretti Gallo, Elza Padilha Ferri, Pamella de Barros Motta, Sandra Kalil Bussadori.

**Writing – review & editing:** Laura Hermida Bruno, Ana Paula Taboada Sobral, Marcela Leticia Leal Gonçalves, Ana Laura Fossati, Elaine Marcilio Santos, Juliana Maria Altavista Sagretti Gallo, Elza Padilha Ferri, Pamella de Barros Motta, Anna Carolina Ratto Tempestini Horliana, Lara Jansiski Motta, Sandra Kalil Bussadori.
